# Information needs of patients with lung cancer from diagnosis until first treatment follow-up

**DOI:** 10.1371/journal.pone.0199515

**Published:** 2018-06-21

**Authors:** Ling-Yu Hsieh, Fang-Ju Chou, Su-Er Guo

**Affiliations:** 1 Department of Nursing, Ditmanson Medical Foundation Chia-Yi Christian Hospital, Chiayi City, Taiwan; 2 Cancer Center, Ditmanson Medical Foundation Chia-Yi Christian Hospital, Chiayi City, Taiwan; 3 Graduate Institute of Nursing & Chronic Diseases and Health Promotion Research Centre, College of Nursing, Chang Gung University of Science and Technology, Puzi City, Chiayi County, Taiwan; 4 Division of Pulmonary and Critical Care Medicine, Chiayi Chang Gung Memorial Hospital, Chang Gung Medical Foundation, Puzi City, Chiayi County, Taiwan; 5 Department of Safety Health and Environmental Engineering, Ming Chi University of Technology, New Taipei City, Taiwan; North Shore Long Island Jewish Health System, UNITED STATES

## Abstract

The aim of this study was to analyze the information needs of lung cancer patients from diagnosis until first treatment follow-up. Sixty-nine participants with lung cancer were recruited from Ditmanson Medical Foundation Chia-Yi Christian Hospital in Midwest Taiwan. The Modified Toronto Informational Needs Questionnaire (TINQ) was used to assess information needs during visits to the outpatient oncology department. Generalized estimating equations were applied to compare changes in information needs over time and to examine correlates of information needs of lung cancer patients. The greatest concern of lung cancer patients was the cancer itself and access to recovery information. The need for information regarding food selection and social welfare resources was also high. However, the means of information needs for each domain significantly decreased over time. Demographic information (age, gender, disease stage, current treatment, education, work status, and having children) was significantly associated with information needs over time. The need for “disease-related information” remained high regardless of disease stage. Oncology nurses can use the results of this study to better address the information needs of patients in an effort to fill knowledge gaps between patients and healthcare providers. Further studies are needed to explore the use of an appropriate instrument, like that used in this study, to identify newly-diagnosed lung cancer patients’ difficulties, concerns, and target interventions to improve their quality of life.

## Introduction

According to the latest statistical data compiled by the Taiwanese Ministry of Health and Welfare, lung cancer is ranked number four among the top 10 most common cancers and currently has the highest death rate [1]. Patients with lung cancer often present with cough, dyspnea, pain, dysphagia, and other general symptoms. Liao et al. reported that patients with lung cancer averaged 10.4 different kinds of symptoms [2], and Krishnasamy, Wilkie, and Haviland [3] indicated that nearly 50% of patients with lung cancer experienced dyspnea, even while resting. However, only 15% of patients with lung cancer have been given guidance on how to cope with the disease [3]. Many patients have expressed the need for information concerning treatment and examination, physical pain, the body’s response to side effects, and the impact on living. If these information needs are not met, patients may feel uncertain or possibly choose to discontinue treatment. As patients obtain more information, their decision-making capacity increases and their response to the disease improves, thereby enhancing their quality of treatment and care, reducing anxiety and emotional distress, and promoting emotional stability with family members [4–5].

Some studies, including a systematic review [6], have demonstrated supportive care needs among patients with lung cancer; however, most studies have not addressed their needs over time [6–8] or just focused on the advanced stage or palliative radiotherapy [9]. Halkett et al. [10] indicated that the information needs of patients differed during various stages of treatment and recommended continually confirming the information needs of patients throughout four stages of treatment: at the initial physician consultation, at the joint development of the follow-up treatment plan, at the initial treatment, and on completion of treatment. Research has indicated that patients tend to desire more extensive information during the initial stage of the disease, with information concerning the disease, treatment, and prognosis being most important to them [5]. However, patients rated psychosocial information as a low concern [11], and throughout treatment, information needs are generally believed to decrease over time. Previous studies have indicated that even after completion of treatment, the need for information remains high. A study by Guleser, Tasci, and Kaplan [12] showed that more than 83% of patients with cancer undergo radiotherapy, and treatment-induced complications lead to the need for additional information. Similarly, Rutten et al. [5] revealed that newly diagnosed patients in the treatment phase needed information on stages of the disease, treatment plans, and treatment-related side effects; yet, patients who completed treatment still expressed a need for information on side effects, rehabilitation, and self-care.

As evidenced by the literature, each treatment stage requires the development of new therapeutic goals, thereby resulting in different patient information needs. In addition, examining information needs over time is important and has been recommended in several studies [7–8]. Therefore, to provide information that actually meets the needs of patients, healthcare personnel should consider the individual needs of patients and make assessments accordingly [13]. While there have been numerous studies on the information needs of patients with gynecologic cancer [14], breast cancer [15–16], gastrointestinal cancer [17], and colorectal cancer [18], there have been only a few studies examining the information needs of patients with lung cancer, with no studies examining the changes and correlates of information needs over time.

The aim of this study was to investigate the information needs of patients with lung cancer from initial diagnosis to first treatment follow-up. Understanding the information needs of patients with lung cancer at each stage of treatment will enable patient-centric consulting services.

## Materials and methods

### Sample and setting

This descriptive longitudinal study was conducted to compare the information needs of patients with lung cancer from initial diagnosis to first treatment follow-up. A convenience sample of patients with lung cancer was recruited from the departments of thoracic surgery, hematology and oncology, and radiation oncology at one teaching hospital in Midwest Taiwan. This study used face-to-face interviews with structured questionnaires to collect patient data from diagnosis (Time 1) to first treatment follow-up (Time 2, approximately 5 months after first treatment). Data were collected from October 2013 to February 2016. A total of 69 patients were included in the study (S1 Dataset). However, two patients were unavailable for data collection at Time 2. Therefore, 67 patients were included in the Time 2 analysis.

The study protocol was approved by the Institutional Review Board at the study hospital (CYCH IRB No: 102035). A signed consent form was obtained from each participant. Participants who met the study criteria were informed of the research purposes, intervention benefits and risks, procedures, and instruments. The inclusion criteria were as follows: (1) patients newly diagnosed with primary lung cancer, (2) patients with sufficient verbal communication skills, and (3) patients aged 20 years or older. The exclusion criteria were as follows: (1) patients with psychiatric disorders, (2) patients who did not know their disease condition, and (3) patients who were in a critical condition.

### Variables and instruments

#### Information needs of patients with lung cancer

The Modified Toronto Informational Needs Questionnaire-Breast Cancer (TINQ-BQ) was originally developed by Galloway et al. (1997) and consists of 52 items from five subscales: disease, investigative tests, treatment, physical care, and psychological needs [19]. The present study used the Chinese version of the Toronto Informational Needs Questionnaires-Breast Cancer (TINQ–BC), revised by Lei et al. [15], which had a good internal consistency reliability rating of 0.99 for the total questionnaire and alpha ratings ranging from 0.87 to 0.97 for each subscale. The questionnaire was validated by professionals in the field of oncology, but some questions from the original survey were not suitable for patients with lung cancer. Therefore, after discussions with clinical care professionals, 17 items that were not applicable were deleted from the original questionnaire and 11 new items were added (S1 and S2 Files). A hematology-oncology nurse practitioner, two senior nurses, a thoracic surgery nurse practitioner, and a cancer social worker were asked to analyze the contents, semantics, and appropriateness of the questionnaire. The resulting content validity index (CVI) score was 0.88, and a pilot study was conducted to confirm that the questionnaire was appropriate and applicable. The final questionnaire consisted of a total of 46 items, including 5 items for disease, 6 items for examination, 15 items for treatment, 7 items for physical care, and 13 items for psychosocial needs. A 5-point Likert scale was used to rate information needs from 1 (not important) to 5 (extremely important), with higher scores indicating a higher degree of need. The internal consistency of the overall scale was found to be favorable (Cronbach’s alpha: 0.95), and the internal consistency (Cronbach’s alpha) of the subscales ranged from 0.75 to 0.94.

#### Demographic data

In the present study, information on age, gender, marital status, education, work status, chronic disease, children (having children or not having children), cancer history, economic satisfaction, disease stage, current treatment, and living situation (alone or with families) was collected for each patient.

### Statistical analysis

All statistical analyses were conducted using SPSS version 21.0 (IBM Corp., Somers, NY, USA) for Windows, and the frequency distribution, percentage, mean, and standard deviation of the basic data of the patients were calculated. The paired samples t-test was performed to evaluate the difference in score between Time 1 and Time 2 with normal distribution and the Wilcoxon signed-rank test was used as the difference score without normal distribution. Furthermore, generalized estimation equations (GEE) were used to investigate changes in information needs for different cancer stages and analyze factors related to information needs of each domain over time. In all of the analyses, statistical significance was defined as *p* < 0.05.

## Results

### Participant characteristics

The mean age of the study subjects was 57.58 ± 9.91 years, with male subjects comprising 56.5%, married subjects comprising 73.9%, subjects with a senior high school certificate or college degree comprising 59.4%, and subjects who were unable to work because of illness comprising 47.8% of the total population. The details of the demographic data are reported in Table 1. The majority of the patients (46.4%) had been diagnosed with stage IV lung cancer, and chemotherapy was the most common treatment method (49.3%).

**Table 1:** Demographic characteristics of the participants (n = 69).

**Variable**	
**Age, year±SD(range)**	57.58±9.91(28~81)
**Gender (%)**	
Male	39(56.5)
Female	30(43.5)
**Marital status (%)**	
Not married	6(8.7)
Married	51(73.9)
Divorced/Widowed	12(17.4)
**Education (%)**	
Illiterate	6(8.7)
Junior high school	22(31.9)
Senior high school	30(43.5)
College degree	11(15.9)
**Work status (%)**	
Homemaker	22(31.9)
Working	14(20.3)
Unable to work because of illness	33(47.8)
**Chronic disease (%)**	
No	39(56.5)
Yes	30(43.5)
**Had children**	
No	7(10.1)
Yes	62(89.9)
**Cancer history**	
No	63(91.3)
Yes	6 (8.7)
**Economic satisfaction**	
Dissatisfied	18(26.1)
Satisfied	51(73.9)
**Disease stage (%)**	
Ⅰ	20(29)
Ⅱ	2(2.9)
Ⅲ	15(21.7)
Ⅳ	32(46.4)
**Current treatment (%)**	
Surgery	20(29)
Chemotherapy	34(49.3)
Targeted therapy	15(21.7)
**Living situation (%)**	
Alone	2(2.9)
With family	67(97.1)

### Changes in information needs from Time 1 to Time 2

The overall mean scores at Time 1 and Time 2 were 3.72 and 2.03, respectively, indicating that patients in the initial stage of lung cancer had high overall information needs that significantly decreased over time. When lung cancer was first diagnosed, disease-related information needs were the highest, with an average score of more than 4 points, followed by physical care-related information. Examination-related information needs had the lowest score. At Time 2, only disease-related information needs remained high, and information needs regarding the other four domains decreased significantly (Table 2). Furthermore, significant differences were observed in all five main domains (*p* < 0.001).

**Table 2:** Comparison of information needs of patients with lung cancer over time.

Subscales	Time 1(N = 69)			Time 2 (N = 67)			
	Mean ±SD	Actual range	Rank	Mean ±SD	Actual range	Rank	P-value
Disease- related	4.42±0.79	1.80–5.00	1	3.27±1.00	1.00–5.00	1	< .001^a^
Physical care- related	3.98±1.03	1.00–5.00	2	1.98±0.89	1.00–5.00	2	< .001^b^
Treatment- related	3.91±1.08	1.13–5.00	3	1.85±0.95	1.00–4.67	4	< .001^b^
Psychosocial- related	3.41±1.02	1.00–5.00	4	1.87±0.80	1.00–4.46	3	< .001^a^
Examination- related	3.03±1.11	1.00–5.00	5	1.85±0.88	1.00–4.33	5	< .001^a^

Possible range of information needs questionnaire is 1–5.

Two cases lost to follow-up at Time 2 were dropped for the paired test.

^a^The paired samples t-test was performed to evaluate the differences in score between Time 1 and Time 2 with normal distribution

^b^The Wilcoxon signed-rank test was used to evaluate the differences in score between Time 1 and Time 2 without normal distribution.

### Information needs at Time 1 and Time 2

In the present study, the top 10 highest information needs at Time 1 received scores of over 4 points, with “how lung cancer acts in the body” receiving the highest score. The questions of “which foods I can or cannot eat,” “if there is any financial support or social welfare available to me during my illness,” and “how much the medical costs will be during my treatment” ranked fourth, sixth, and tenth, respectively. At Time 2, the question of “if the lung cancer will come back” also had a mean score over 4 points. The questions of “if there is cancer anywhere else in my body,” and “how to know if the cancer has come back” received scores of over 3.5 points. However, the mean score of the other items dropped to 2–3 points. The lowest scored items were examination-related and psychosocial needs. Detailed information is presented in Table 3.

**Table 3:** Top 10 highest and lowest information needs at Time 1 and Time 2.

Items	Time 1 (N = 69)			Time 2 (N = 67)		
	Mean	SD	Rank	Mean	SD	Rank
***Highest***						
**Disease-related**						
How lung cancer acts in the body	4.70	0.75	1	3.15	1.70	5
How to know the cancer has come back	4.65	0.74	2	3.52	1.63	3
If the lung cancer will come back	4.51	1.09	3	4.12	1.32	1
If there is cancer anywhere else in my body				3.85	1.44	2
**Physical care-related**						
Which foods I can or cannot eat	4.42	1.25	4	3.19	1.68	4
How to take care myself after the treatment	4.41	1.22	5	2.58	1.69	9
If there are any physical things I should not do	4.19	1.31	9			
**Psychosocial-related**						
If there is any financial support or social welfare available to me during my illness	4.33	1.16	6	3.04	1.69	6
How the illness may affect my life in the future	4.26	1.24	7	2.67	1.68	8
How much the medical cost during my treatment	4.17	1.28	10			
**Treatment-related**						
When the target therapy are done	4.20	1.34	8	2.39	1.83	10
**Examination-related**						
When the tests are done again				2.91	1.78	7
***Lowest***						
**Examination-related**						
How the tests are done	2.19	1.50	1	1.43	0.96	5
How I will feel during the tests	2.22	1.46	2			
The reason the doctor suggests certain tests	3.03	1.71	8			
Why they often need to test my blood	3.20	1.69	10			
**Psychosocial-related**						
How to talk to family/friends about illness	2.55	1.46	3	1.52	1.06	9
What to do if I feel uncomfortable in social situations	2.67	1.49	4	1.40	0.84	4
What to do if I become concerned about dying	2.70	1.58	5			
If I will need help taking care of myself	2.81	1.49	6	1.40	0.89	3
If I can continue with my usual social activities	2.96	1.59	7	1.48	1.01	8
Where I can get help to deal with my feelings about my illness	3.10	1.60	9			
**Treatment-related**						
Why I need to insert Port-A-cath				1.36	0.98	1
How the Port-A-cath is done				1.46	1.12	7
**Physical care-related**						
How long will my wound take to heal				1.37	0.87	2
How long my wound can run into the water				1.46	1.02	6
If I need to do pulmonary rehabilitative exercise after surgery				1.57	1.14	10

### Factors influencing information needs over time

After adjustment for patient demographic factors, information needs significantly decreased within each domain over time (Table 4). GEE analysis revealed that disease stage, current treatment, age, gender, education, work status, and having children were significantly associated with information needs within a specific domain over time. Within the examination-related domain, patients diagnosed with stage III lung cancer had higher-rated information needs than patients diagnosed with stage I and II lung cancer (estimate: 0.73, p = 0.032). There was an interaction between disease stage and time, indicating that patients with stage IV lung cancer had significant increases from Time 1 to Time 2 regarding needs for information compared to patients with stage I and II lung cancer within the disease- and physical care-related domains (estimate: 0.84 and 0.69, p = 0.016 and 0.033, respectively).

**Table 4:** Factors influencing each domain of information needs by GEE analysis.

Variable	Disease-related			Physical-related			Treatment-related			Psychosocial-related			Examination-related		
	Estimate	SE	p	Estimate	SE	p	Estimate	SE	p	Estimate	SE	p	Estimate	SE	p
**Intercept**	2.72	0.60	<0.001	2.26	0.78	0.004	1.29	0.80	0.108	1.38	0.71	0.054	1.97	0.83	0.018
**Time**															
**Time 1**	ref			ref			ref			ref			ref		
**Time 2**	-1.65	0.26	<0.001	-2.42	0.24	<0.001	-2.23	0.24	<0.001	-1.67	0.19	<0.001	-1.16	0.35	0.001
**Disease stage**															
**I&II**	ref			ref			ref			ref			ref		
**III**	0.28	0.26	0.275	0.47	0.35	0.173	0.58	0.36	0.102	0.32	0.33	0.337	0.73	0.34	0.032
**IV**	-0.25	0.32	0.433	0.00	0.37	1.000	0.58	0.38	0.124	0.52	0.35	0.137	0.20	0.40	0.615
Time*Disease stage^**a**^															
**I&II**	ref			ref			ref			ref			ref		
**III**	0.50	0.39	0.197	0.47	0.41	0.251	0.37	0.39	0.350	0.66	0.35	0.061	-0.10	0.50	0.836
**IV**	0.84	0.35	0.016	0.69	0.32	0.033	0.23	0.33	0.489	-0.01	0.27	0.978	0.07	0.41	0.875
**Current treatment**															
**Surgery**	ref			ref			ref			ref			ref		
**Chemotherapy**	-0.14	0.27	0.617	-0.50	0.23	0.028	-0.02	0.26	0.936	-0.30	0.25	0.236	0.14	0.25	0.563
**Targeted therapy**	-0.04	0.30	0.896	-0.34	0.35	0.328	-0.11	0.36	0.758	-0.10	0.31	0.747	0.19	0.35	0.583
**Age**	0.02	0.01	0.033	0.02	0.01	0.045	0.03	0.01	0.006	0.02	0.01	0.043	0.01	0.01	0.461
**Gender**															
**Male**	ref			ref			ref			ref			ref		
**Female**	-0.05	0.17	0.778	0.20	0.20	0.313	0.42	0.22	0.049	0.25	0.15	0.097	-0.28	0.17	0.106
**Marital status**															
**Not married**	ref			ref			ref			ref			ref		
**Married**	0.01	0.38	0.983	-0.13	0.49	0.784	0.22	0.56	0.688	-0.40	0.42	0.341	-0.17	0.50	0.738
**Divorced /widowed**	-0.23	0.29	0.436	-0.23	0.34	0.495	-0.21	0.47	0.654	-0.49	0.33	0.140	-0.27	0.42	0.523
**Education**															
**Illiterate**	ref			ref			ref			ref			ref		
**Junior high school degree**	-0.21	0.23	0.363	0.23	0.34	0.493	0.31	0.32	0.331	0.15	0.26	0.579	-0.08	0.31	0.799
**Senior high school**	0.11	0.19	0.552	0.34	0.32	0.284	0.48	0.33	0.146	0.44	0.29	0.136	-0.09	0.31	0.765
**College degree**	0.20	0.16	0.224	0.44	0.32	0.177	0.21	0.39	0.582	0.68	0.29	0.019	-0.02	0.33	0.959
**Work status**															
**Homemaker**	ref			ref			ref			ref			ref		
**Working**	0.47	0.26	0.070	0.16	0.27	0.550	0.22	0.23	0.333	0.14	0.22	0.526	-0.07	0.23	0.755
**Unable to work because of illness**	0.20	0.17	0.237	0.51	0.18	0.005	0.55	0.20	0.007	0.75	0.19	<0.001	-0.03	0.18	0.848
**Economic satisfaction**															
**Dissatisfied**	ref			ref			ref			ref			ref		
**Satisfied**	-0.11	0.17	0.503	-0.02	0.24	0.929	0.02	0.22	0.939	-0.05	0.20	0.795	0.26	0.19	0.157
**Children**															
**No**	ref			ref			ref			ref			ref		
**Yes**	0.77	0.28	0.005	0.13	0.33	0.685	-0.35	0.32	0.266	0.45	0.27	0.096	0.46	0.28	0.099
**Cancer history**															
**No**	ref			ref			ref			ref			ref		
**Yes**	-0.12	0.21	0.563	0.20	0.29	0.486	0.11	0.34	0.738	0.31	0.32	0.333	0.17	0.26	0.513

GEE, generalized estimation equation; SE, standard error; ref, reference group.

^a^ Based on Time 2 compared to Time 1, lung cancer stages III and IV compared to stages I and II

Within the physical care-related domain, patients with current chemotherapy treatment had lower information needs than surgery (estimate: 0.50, p = 0.028). Older patients had higher-rated information needs than those of younger patients within each domain, with the exception of examination-related information. Within the treatment-related domain, women had higher-rated information needs than men (estimate: 0.42, p = 0.049). Within the psychosocial-related domain, patients with higher education levels had higher-rated information needs than patients with lower education levels (college degree compared to illiterate, estimate: 0.68, p = 0.019). Within the physical care-, treatment-, and psychosocial-related domains, patients who were unable to work because of illness had higher information needs than patients who were homemakers (estimates: 0.51, 0.55, and 0.75, respectively; p<0.01 each). Within the disease-related domain, patients who had children had higher-rated information needs than those with no children (estimate: 0.77, p = 0.005). Marital status, economic satisfaction, and cancer history were factors not significantly associated with any domain.

## Discussion

To the best of our knowledge, this study is the first prospective longitudinal study that focuses on examining the information needs of patients with lung cancer from initial diagnosis until first treatment follow-up. Information about patients’ needs is crucial for developing appropriate interventions to improve their quality of life. This study addressed a gap in the existing literature by exploring the information needs of Chinese patients with lung cancer, particularly with regard to the period from the initial diagnosis until first treatment follow-up. We also aimed to examine the correlates of information needs over time among the patients.

The results revealed that at initial diagnosis, the overall information needs of the patients were high. Disease-related information needs ranked first, while physical care-related information needs ranked second. This was consistent with the results of previous studies that showed that psychological and physical care needs were mostly in the domains of concerns or unmet social needs [6, 8]. The items commonly identified in the present study are recurrence and worry about cancer spread, which are understandable and similar to those identified in previous studies that focused on lung cancer and ovarian cancer patients [8, 20]. However, some items were slightly different. A cross-sectional study conducted by Giuliani et al. [8] assessed supportive care needs among survivors of any stage of lung cancer, and indicated that the psychological domains of anxiety and depression were the most prevalent unmet care needs (66%) in this population. In previous studies, psychological domains have been reported to be the most common unmet needs among patients with various types of cancer, including lung cancer [21]. That may likely be the result of our study; however, we only explored information needs, not unmet needs which are slightly different. Understanding patients’ unmet needs is important to develop best practices and to direct resources to address those needs; however, it does not portray the entirety of patients’ needs and priorities. Further studies are needed to examine information needs, unmet needs, and “want help needs” and to understand and recognize the overall needs of lung cancer patients in order to address and meet those needs. In addition to reporting the ranking of unmet needs, Giuliani et al. [8] also reported information needs with regard to symptoms management, which serves as evidence to healthcare providers to establish appropriate interventions. However, we were unable to explore those needs in the present study; this should be investigated in future studies.

In addition, at the initial treatment follow-up, patient information needs had significantly decreased, with the exception of disease-related information needs, which remained comparatively high. Age, gender, disease stage, current treatment, education, work status, and having children were significantly associated with information needs in multiple subscales over time.

During the initial stage of disease, patients often desire information regarding various concerns to better understand and cope with their disease. However, over time, the information needs of patients tend to decrease, indicating their ability to receive and process information throughout the treatment process [4,22]. In the present study, patient information needs decreased over time, with the exception of disease-related information needs, and this finding is consistent with the results of three previous studies [4,15,23]. Since patients with lung cancer often undergo several treatments, information needs change over time, which can explain the observed trend in the present study. In addition, given the uncertainty that surrounds the pathology of lung cancer, many patients remain anxious about the prognosis and recurrence of the disease, even after completing treatment. Lei et al. [15] examined information needs of patients with breast cancer in the first and fourth chemotherapy cycles, and found that in both cycles, the top three concerns were whether or not cancer cells were present in other parts of the body, how to identify cancer recurrence, and whether or not the breast cancer would recur. Similarly, Mistry et al. [22] surveyed the information needs of 187 patients with cancer at both the pre- and post-treatment stages, and found that 90% of the subjects had a high degree of information needs regarding the prognosis of the disease, including issues related to the probability of recurrence and metastasis.

The present study found that patients were extremely concerned about their choices of food at both Time 1 and Time 2. Lei et al. [15] and O'Connor et al. [18] also found information on food choices to be a concern of patients. Therefore, information on nutrition should be provided to patients at different stages of disease and treatment. Further, the Formosa Cancer Foundation 2005 Cancer Patient Nutrition Awareness Survey revealed that patients and their family members have misconceptions about nutrition. Forty percent of the respondents incorrectly believed that the “intake of too much nutrition (would) make the tumor grow faster,” and almost 80% indicated a need for more information on nutrition [24]. Thus, medical professionals should provide patients with sufficient information on food choices and dietary habits.

Our data showed that the patients were quite concerned about information on social welfare resources and treatment costs. Similarly, Luo, Yu, and Hsieh [25] found that patients on hemodialysis had an increased need for information on social welfare and medical subsidies throughout the treatment process. Since 50% of the patients in the present study were unable to continue working because of disease-related factors, there was an increase in medical expenses at a time when the household income suddenly decreased, often leaving the family with a heavy financial burden. Therefore, information on proposed medical subsidies and related benefits from the hospital, government, and nonprofit organizations should be included in the health education provided to patients. Conversely, the present study found that patients had a low need for psychosocial information during all stages of treatment, which is consistent with previous studies [4,11]. This may be the result of patients still focusing on the disease itself and not on the emotional implications of the disease [15].

The present study identified several sociodemographic characteristics that were associated with patient information needs within multiple domains. Age (older), gender (women), disease stage (stage III), education (higher), work status (unable to work because of illness), and having children were significantly associated with higher information needs within specific domains. Similarly, Mistry et al. [22] reported that age (older) and gender (women) were significant predictors of increased information needs within multiple domains. Matsuyama et al. [4] also reported that women were more likely to have higher information needs within multiple domains. Unlike the present study, both studies identified individuals with lower educational statuses to be more likely to have higher information needs [4,22]. Differences between the results of the present study and previous studies could be attributed to cultural differences. Cultural perspectives influence attitudes toward illnesses and treatments [26]. For example, in Taiwan, individuals with lower education levels tend to obey physician authority, and adhere to the suggested treatment [27]. Further, the present study revealed that patients with college education had more psychosocial-related information needs than patients without college education (Table 4).

In addition, the present study found that patients with stage III lung cancer had more examination-related needs than patients with stage I and II, consistent with Giuliani et al. [8]; however, this contradicts Matsuyama et al. [4], who found no significant associations between disease stages. While stage I and stage II lung cancer are often treated by surgical resection, stage III cancer is typically treated with chemotherapy and radiotherapy. Therefore, patients with stage III lung cancer must regularly undergo laboratory and imaging tests to monitor the effectiveness of those treatments. Healthcare providers should ensure patients with late-stage cancer have the necessary psychological and examination-related information. Further, healthcare providers should be attentive to patient information needs and regularly inquire about patient information needs and preferences throughout the treatment process.

The present study had several limitations. First, the sample size was limited to patients with lung cancer at a single institution; however, the sample, which contained more men and patients diagnosed with stage Ⅳ lung cancer, was representative of the national profile based on the results of the Taiwanese Ministry of Health and Welfare [1]. Second, although the scale of information needs utilized in the present study had good reliability and content validity, there were too many items, which may have influenced the willingness of the subjects to contribute. Therefore, a simplified version of the questionnaire should be developed in the future.

### Implications for nursing

While the information needs of the patients changed over time, the patients maintained a high need for information concerning lung cancer recurrence and metastasis. Patients also expressed a high need for information on food selection and social welfare resources. These results indicate that the communication between healthcare providers and patients at Time 2 is not sufficient, which is consistent with the findings of Ugalde, Aranda, Krishnasamy, Ball, and Schofield, [28]. Communication between healthcare providers and patient needs to improve, specifically within disease-related and physical care-related domains, to meet the most urgent needs of patients with lung cancer. Healthcare providers must understand the individual needs of patients with lung cancer to develop tailored, patient-centered educational programs.

Age, gender, disease stage, education, work status, and having children were found to be significantly associated with information needs within multiple subscales over time; thus, clinical healthcare providers should consider these factors and prepare structured and culturally appropriate content when communicating medical information to patients with lung cancer.

## Conclusions

The information needs of patients with lung cancer change over time. Medical communication between healthcare providers and patients with lung cancer needs to be improved, with nurses playing an active role in the development of informative, patient-centric education programs. The results of this longitudinal study provide additional insight into the patterns of information that patients with lung cancer seek. However, a more detailed, multidisciplinary analysis needs to be conducted to further understand whether or not healthcare providers, including nurses, meet patients’ information needs/unmet needs and what kinds of resources patients need to better obtain and process this information.

## Supporting information

S1 File Patient Informational Needs Questionnaire. (DOC)

S2 File Patient Informational Needs Questionnaire (Chinese Vesion). (DOC)

S1 Dataset Data information. (PDF)
