# Supplementary material for: Information needs of patients with lung cancer from diagnosis until first treatment follow-up
Source: PLoS One. 2018 Jun 21;13(6):e0199515. doi: 10.1371/journal.pone.0199515 (PMC6013211; doi:10.1371/journal.pone.0199515)
Supplement: S1 File — (DOC) [file pone.0199515.s001.doc]

**Patient Informational Needs Questionnaire**

**What information do you need during treatment？ A 5-point Likert scale was used to rate the information needs.**

1 ＝ extremely not need

2 ＝ not need 3 ＝ passably

4 ＝ need

5 ＝ extremely need

| **Item 1~15** | | | | | | |
| --- | --- | --- | --- | --- | --- | --- |
| 1 | How I will feel during the tests | 1 | 2 | 3 | 4 | 5 |
| 2 | How the tests are done | 1 | 2 | 3 | 4 | 5 |
| 3 | The reason the doctor suggests certain tests | 1 | 2 | 3 | 4 | 5 |
| 4 | When the tests are done again | 1 | 2 | 3 | 4 | 5 |
| 5 | Why they often need to test my blood | 1 | 2 | 3 | 4 | 5 |
| 6 | What does the blood test report mean? | 1 | 2 | 3 | 4 | 5 |
| 7 | If there is cancer anywhere else in my body | 1 | 2 | 3 | 4 | 5 |
| 8 | If the lung cancer will come back | 1 | 2 | 3 | 4 | 5 |
| 9 | How lung cancer acts in the body | 1 | 2 | 3 | 4 | 5 |
| 10 | How to know the cancer has come back | 1 | 2 | 3 | 4 | 5 |
| 11 | Can my lung cancer be inherited? | 1 | 2 | 3 | 4 | 5 |
| 12 | Why I need to insert Port-A-cath | 1 | 2 | 3 | 4 | 5 |
| 13 | How the Port-A-Cath is done | 1 | 2 | 3 | 4 | 5 |
| 14 | When Port-A-Cath can be removed | 1 | 2 | 3 | 4 | 5 |
| 15 | How Chemotherapy is to proceed | 1 | 2 | 3 | 4 | 5 |

1 ＝ extremely not need

2 ＝ not need 3 ＝ passably

4 ＝ need

5 ＝ extremely need

| **Item 16~28** | | | | | | |
| --- | --- | --- | --- | --- | --- | --- |
| 16 | How to prepare before chemotherapy | 1 | 2 | 3 | 4 | 5 |
| 17 | How long does chemotherapy take? | 1 | 2 | 3 | 4 | 5 |
| 18 | If chemotherapy will change my appearance | 1 | 2 | 3 | 4 | 5 |
| 19 | Will chemotherapy weaken my immune system and make more prone to infection | 1 | 2 | 3 | 4 | 5 |
| 20 | What types of side effects I will have after chemotherapy | 1 | 2 | 3 | 4 | 5 |
| 21 | Who should consult if I have questions during chemotherapy | 1 | 2 | 3 | 4 | 5 |
| 22 | Is there any way to avoid the side effects of chemotherapy? | 1 | 2 | 3 | 4 | 5 |
| 23 | Why do doctors recommend this treatment | 1 | 2 | 3 | 4 | 5 |
| 24 | When the target therapy are done | 1 | 2 | 3 | 4 | 5 |
| 25 | Who should I consult if I heard other treatment except surgery, chemotherapy, radiotherapy | 1 | 2 | 3 | 4 | 5 |
| 26 | What are the most possible side effects of chemotherapy, targeted therapies, radiotherapy and surgery? | 1 | 2 | 3 | 4 | 5 |
| 27 | How to take care of Port-A-Cath | 1 | 2 | 3 | 4 | 5 |
| 28 | *If there is any physical thing I should not do* | 1 | 2 | 3 | 4 | 5 |

1 ＝ extremely not need

2 ＝ not need 3 ＝ passably

4 ＝ need

5 ＝ extremely need

| **Item 29~44** | | | | | | |
| --- | --- | --- | --- | --- | --- | --- |
| 29 | How long my wound can run into the water | 1 | 2 | 3 | 4 | 5 |
| 30 | How long will my wound take to heal | 1 | 2 | 3 | 4 | 5 |
| 31 | If I need to do pulmonary rehabilitation exercise after surgery | 1 | 2 | 3 | 4 | 5 |
| 32 | Which foods I can or cannot eat | 1 | 2 | 3 | 4 | 5 |
| 33 | How to take care myself after the treatment | 1 | 2 | 3 | 4 | 5 |
| 34 | How the illness may affect my life in the future | 1 | 2 | 3 | 4 | 5 |
| 35 | Where my family can get help while taking care of me | 1 | 2 | 3 | 4 | 5 |
| 36 | If the illness may affect me and my family life | 1 | 2 | 3 | 4 | 5 |
| 37 | What to do if I become concerned about dying | 1 | 2 | 3 | 4 | 5 |
| 38 | If I can continue to my hobby and exercise | 1 | 2 | 3 | 4 | 5 |
| 39 | Where I can get help to deal with my feelings about my illness | 1 | 2 | 3 | 4 | 5 |
| 40 | How to talk to family/friends about illness | 1 | 2 | 3 | 4 | 5 |
| 41 | If I will need help taking care of myself | 1 | 2 | 3 | 4 | 5 |
| 42 | What to do if I feel uncomfortable in social situations | 1 | 2 | 3 | 4 | 5 |
| 43 | If I can continue with my usual social activities | 1 | 2 | 3 | 4 | 5 |
| 44 | Is there any course of lung cancer | 1 | 2 | 3 | 4 | 5 |

1 ＝ extremely not need

2 ＝ not need 3 ＝ passably

4 ＝ need

5 ＝ extremely need

| **Item 45~46** | | | | | | |
| --- | --- | --- | --- | --- | --- | --- |
| 45 | If there is any financial support or social welfare available to me during my illness | 1 | 2 | 3 | 4 | 5 |
| 46 | How much the medical cost during my treatment | 1 | 2 | 3 | 4 | 5 |
