# Supplementary material for: Information needs of patients with lung cancer from diagnosis until first treatment follow-up
Source: PLoS One. 2018 Jun 21;13(6):e0199515. doi: 10.1371/journal.pone.0199515 (PMC6013211; doi:10.1371/journal.pone.0199515)
Supplement: S2 File — (DOC) [file pone.0199515.s002.doc]

**病人訊息需求量表**

**在進行治療這段期間，您最需要哪些訊息？ 請於1至5表示訊息的需要程度。（每題只圈出一個號碼而已）**

1 ＝ 非常不需要

2 ＝ 不需要 3 ＝ 尚可

4 ＝ 需要

5 ＝ 非常需要

| **關於以下的資訊，您的需求為何?** | | | | | | |
| --- | --- | --- | --- | --- | --- | --- |
| 1 | 當進行斷層掃描、核磁共振和骨頭掃描時我會有什麼感覺 | 1 | 2 | 3 | 4 | 5 |
| 2 | 斷層掃描、核磁共振、骨頭掃描是如何進行的 | 1 | 2 | 3 | 4 | 5 |
| 3 | 醫生建議進行的斷層掃瞄、核磁共振、骨頭掃描之用意是什麼 | 1 | 2 | 3 | 4 | 5 |
| 4 | 何時需再進行斷層掃描、核磁共振、骨頭掃描 | 1 | 2 | 3 | 4 | 5 |
| 5 | 為何需要常常抽血 | 1 | 2 | 3 | 4 | 5 |
| 6 | 抽血報告代表什麼意思 | 1 | 2 | 3 | 4 | 5 |
| 7 | 我身體其他部分會有癌症細胞嗎 | 1 | 2 | 3 | 4 | 5 |
| 8 | 肺癌是否會復發 | 1 | 2 | 3 | 4 | 5 |
| 9 | 肺癌對我的身體會造成什麼影響 | 1 | 2 | 3 | 4 | 5 |
| 10 | 如何知道癌症復發 | 1 | 2 | 3 | 4 | 5 |
| 11 | 我的癌症是否會遺傳 | 1 | 2 | 3 | 4 | 5 |
| 12 | 為什麼要裝置人工血管 | 1 | 2 | 3 | 4 | 5 |
| 13 | 人工血管裝置是要如何進行 | 1 | 2 | 3 | 4 | 5 |
| 14 | 人工血管何時可以移除 | 1 | 2 | 3 | 4 | 5 |
| 15 | 化療是怎樣進行的 | 1 | 2 | 3 | 4 | 5 |
| 16 | 我應該如何做好化療前的準備 | 1 | 2 | 3 | 4 | 5 |

1 ＝ 非常不需要

2 ＝ 不需要

3 ＝ 尚可

4 ＝ 需要

5 ＝ 非常需要

| **關於以下的資訊，您的需求為何?** | | | | | | |
| --- | --- | --- | --- | --- | --- | --- |
| 17 | 化療療程需要多久 | 1 | 2 | 3 | 4 | 5 |
| 18 | 化療是否會改變我的外貌 | 1 | 2 | 3 | 4 | 5 |
| 19 | 化療會讓我的抵抗力降低而容易受到感染嗎 | 1 | 2 | 3 | 4 | 5 |
| 20 | 進行化療後，我將會有什麼副作用 | 1 | 2 | 3 | 4 | 5 |
| 21 | 化療期間若有疑問，應該向誰諮詢 | 1 | 2 | 3 | 4 | 5 |
| 22 | 是否有避免化療副作用的方法 | 1 | 2 | 3 | 4 | 5 |
| 23 | 醫生為何建議這種治療方式 | 1 | 2 | 3 | 4 | 5 |
| 24 | 標靶治療何時可以進行 | 1 | 2 | 3 | 4 | 5 |
| 25 | 假如聽到手術、化療、放療以外的療法，我應該向誰諮詢 | 1 | 2 | 3 | 4 | 5 |
| 26 | 化療、標靶治療、放射線治療及手術最可能出現的副作用是什麼 | 1 | 2 | 3 | 4 | 5 |
| 27 | 平時如何照顧人工血管 | 1 | 2 | 3 | 4 | 5 |
| 28 | 是否有任何不能進行的動作或活動 | 1 | 2 | 3 | 4 | 5 |
| 29 | 手術後傷口多久可以碰水 | 1 | 2 | 3 | 4 | 5 |
| 30 | 我的手術傷口需要多久才會痊癒. | 1 | 2 | 3 | 4 | 5 |
| 31 | 手術後是否需要做肺部復健運動 | 1 | 2 | 3 | 4 | 5 |
| 32 | 什麼食物可以吃或哪些不能 | 1 | 2 | 3 | 4 | 5 |
| 33 | 治療結束後要如何自我照顧 | 1 | 2 | 3 | 4 | 5 |
| 34 | 此疾病對我日後生活的影響。 | 1 | 2 | 3 | 4 | 5 |
| 35 | 在照顧我的過程中，如果家人需要幫忙，他們可以找誰。 |  |  |  |  |  |

1 ＝ 非常不需要

2 ＝ 不需要 3 ＝ 尚可

4 ＝ 需要

5 ＝ 非常需要

| **關於以下的資訊，您的需求為何?** | | | | | | |
| --- | --- | --- | --- | --- | --- | --- |
| 36 | 是否會改變我和家人的日常生活 | 1 | 2 | 3 | 4 | 5 |
| 37 | 假如我想到死亡，我應該做些什麼。 | 1 | 2 | 3 | 4 | 5 |
| 38 | 是否可以繼續進行我本來的興趣和運動。 | 1 | 2 | 3 | 4 | 5 |
| 39 | 情緒不定時，我如何尋找幫助。 | 1 | 2 | 3 | 4 | 5 |
| 40 | 怎樣和我的家人/朋友談起我的病情。 | 1 | 2 | 3 | 4 | 5 |
| 41 | 生病之後是否需要別人來照顧自己。 | 1 | 2 | 3 | 4 | 5 |
| 42 | 如果跟別人互動時我感覺不自在，我應該怎樣做。 | 1 | 2 | 3 | 4 | 5 |
| 43 | 是否可以進行日常的聚會活動。 | 1 | 2 | 3 | 4 | 5 |
| 44 | 是否有肺癌相關課程 | 1 | 2 | 3 | 4 | 5 |
| 45 | 是否能申請社會福利補助或有相關的福利資源。 | 1 | 2 | 3 | 4 | 5 |
| 46 | 治療過程中所需要的費用。 | 1 | 2 | 3 | 4 | 5 |
