# Supplementary material for: Information needs of patients with lung cancer from diagnosis until first treatment follow-up
Source: PLoS One. 2018 Jun 21;13(6):e0199515. doi: 10.1371/journal.pone.0199515 (PMC6013211; doi:10.1371/journal.pone.0199515)
Supplement: S1 Dataset — (PDF) [file pone.0199515.s003.pdf]

age	gender	marital_status	education	had_children	disease_stage
73	male	married	College degree	yes	III
58	male	married	senior high school	yes	III
55	female	married	senior high school	yes	IV
40	female	married	College degree	yes	IV
64	male	married	College degree	yes	IV
75	male	married	senior high school	yes	IV
50	female	married	Junior high school	yes	IV
81	male	married	Junior high school	yes	I
54	male	married	senior high school	yes	IV
61	male	married	Junior high school	yes	I
53	female	married	Junior high school	yes	III
65	female	divorced/widowed	College degree	yes	IV
49	male	married	senior high school	yes	III
46	male	married	Junior high school	yes	IV
56	male	married	senior high school	yes	III
64	male	married	Junior high school	yes	I
42	female	married	senior high school	yes	IV
56	female	married	Illiterate	yes	IV
59	male	married	Junior high school	yes	III
52	male	not married	Junior high school	no	III
55	female	divorced/widowed	senior high school	yes	IV
69	male	married	senior high school	yes	I
52	female	married	senior high school	yes	I
28	male	not married	senior high school	no	III
71	male	married	College degree	yes	I
40	female	married	Junior high school	yes	I
47	male	divorced/widowed	senior high school	yes	IV
64	male	married	senior high school	yes	III
43	male	divorced/widowed	senior high school	yes	IV
60	female	married	Junior high school	yes	III
57	female	divorced/widowed	Junior high school	yes	IV
65	male	married	Junior high school	yes	III
61	female	married	Illiterate	yes	IV
48	male	divorced/widowed	senior high school	no	IV
66	male	married	Junior high school	yes	I
72	male	married	Illiterate	yes	I
78	male	married	Junior high school	yes	II
66	female	divorced/widowed	Junior high school	yes	IV
46	male	married	Junior high school	yes	IV
72	male	married	Junior high school	yes	IV
43	female	married	senior high school	yes	I
67	female	divorced/widowed	Illiterate	yes	I
45	female	married	senior high school	yes	I
61	male	married	College degree	yes	IV
58	male	married	senior high school	yes	IV
54	male	divorced/widowed	senior high school	yes	IV
59	female	divorced/widowed	senior high school	yes	IV
61	male	married	Junior high school	yes	I
55	female	divorced/widowed	College degree	yes	I
57	female	married	Junior high school	yes	IV
49	female	divorced/widowed	Junior high school	yes	I
64	female	married	senior high school	yes	III

50	male	married	senior high school	yes	IV
74	male	married	College degree	yes	I
58	male	married	Illiterate	yes	IV
49	female	not married	senior high school	no	IV
55	male	married	senior high school	yes	II
54	male	married	senior high school	yes	I
62	female	married	senior high school	yes	I
60	female	married	senior high school	yes	I
57	female	married	Illiterate	yes	IV
51	female	married	Junior high school	yes	IV
52	female	married	senior high school	yes	IV
63	male	married	College degree	yes	III
51	male	not married	senior high school	no	III
65	female	not married	College degree	no	IV
60	female	not married	College degree	no	I
67	male	married	Junior high school	yes	III
59	male	married	senior high school	yes	IV

work_status	economic_satisfaction	chronic_disease	cancer_history
Homemaker	satisfied	no	yes
Unable to work beca	satisfied	no	no
Working	satisfied	no	no
Unable to work beca	satisfied	no	no
Unable to work beca	satisfied	yes	no
Homemaker	satisfied	yes	no
Working	satisfied	no	no
Unable to work beca	satisfied	yes	no
Unable to work beca	satisfied	yes	no
Unable to work beca	satisfied	yes	yes
Homemaker	Dissatisfied	no	no
Homemaker	satisfied	no	no
Unable to work beca	Dissatisfied	no	no
Unable to work beca	Dissatisfied	no	no
Unable to work beca	satisfied	yes	no
Unable to work beca	Dissatisfied	yes	no
Unable to work beca	satisfied	no	no
Unable to work beca	satisfied	yes	no
Unable to work beca	satisfied	no	no
Unable to work beca	Dissatisfied	yes	no
Unable to work beca	satisfied	yes	yes
Unable to work beca	satisfied	yes	no
Working	satisfied	yes	no
Unable to work beca	Dissatisfied	no	no
Homemaker	satisfied	yes	no
Working	satisfied	no	no
Unable to work beca	Dissatisfied	no	no
Homemaker	Dissatisfied	yes	no
Homemaker	Dissatisfied	yes	no
Unable to work beca	Dissatisfied	yes	no
Homemaker	satisfied	yes	no
Unable to work beca	satisfied	yes	no
Unable to work beca	satisfied	yes	no
Unable to work beca	satisfied	no	no
Unable to work beca	Dissatisfied	no	no
Homemaker	satisfied	no	no
Unable to work beca	satisfied	no	no
Homemaker	satisfied	yes	no
Working	satisfied	no	no
Homemaker	satisfied	no	no
Homemaker	satisfied	no	no
Homemaker	satisfied	no	no
Working	Dissatisfied	no	yes
Unable to work beca	satisfied	yes	no
Homemaker	satisfied	no	no
Unable to work beca	Dissatisfied	no	no
Unable to work beca	Dissatisfied	no	no
Unable to work beca	satisfied	no	no
Working	Dissatisfied	no	no
Unable to work beca	Dissatisfied	no	no
Unable to work beca	Dissatisfied	yes	no
Homemaker	satisfied	yes	no

Working	satisfied	yes	yes
Homemaker	satisfied	no	no
Homemaker	satisfied	no	no
Working	satisfied	no	no
Unable to work beca	satisfied	yes	no
Unable to work beca	satisfied	yes	no
Working	satisfied	no	no
Working	satisfied	yes	no
Working	satisfied	yes	no
Working	satisfied	no	no
Homemaker	satisfied	no	yes
Homemaker	satisfied	yes	no
Homemaker	Dissatisfied	no	no
Unable to work beca	satisfied	no	no
Homemaker	satisfied	no	no
Working	satisfied	yes	no
Homemaker	satisfied	no	no

current_treatment	living_situation	Q1	Q2	Q3	Q4
Chemotherapy	with family	5	5	5	5
Chemotherapy	with family	3	3	3	4
Targeted therapy	with family	1	2	5	5
Chemotherapy	with family	3	5	5	3
Chemotherapy	with family	2	2	2	3
Chemotherapy	with family	4	1	4	4
Chemotherapy	with family	5	5	5	5
Chemotherapy	with family	1	1	4	5
Targeted therapy	with family	2	2	2	4
surgery	with family	5	5	5	5
Chemotherapy	with family	1	5	5	5
Targeted therapy	alone	1	1	5	5
Chemotherapy	with family	3	3	5	5
Chemotherapy	with family	1	1	1	1
surgery	with family	3	3	5	4
Chemotherapy	with family	4	1	4	5
Targeted therapy	with family	3	3	3	3
Targeted therapy	with family	4	4	5	5
Chemotherapy	with family	5	5	5	5
Chemotherapy	with family	1	1	1	1
Targeted therapy	with family	4	3	4	2
surgery	with family	1	1	1	5
Chemotherapy	with family	1	1	1	1
surgery	with family	3	3	3	3
surgery	with family	2	2	2	4
surgery	with family	1	1	1	1
Chemotherapy	with family	3	3	3	3
surgery	with family	2	2	2	5
Chemotherapy	with family	1	1	1	3
Chemotherapy	with family	5	5	5	5
Targeted therapy	with family	1	1	1	1
Chemotherapy	with family	1	1	5	5
Targeted therapy	with family	3	3	3	3
Chemotherapy	with family	1	1	1	5
surgery	with family	1	1	1	5
surgery	with family	4	4	4	5
Chemotherapy	with family	1	1	4	5
Targeted therapy	with family	3	2	4	5
Chemotherapy	with family	3	2	4	5
Chemotherapy	with family	3	3	4	5
surgery	with family	1	5	5	5
surgery	with family	3	3	4	5
surgery	with family	1	1	1	4
Chemotherapy	with family	1	1	4	5
Chemotherapy	with family	1	1	5	5
Chemotherapy	with family	2	1	5	5
Targeted therapy	with family	1	1	1	1
Chemotherapy	with family	5	5	5	5
surgery	with family	1	1	1	1
Targeted therapy	with family	1	1	3	5
surgery	with family	1	1	1	1
Chemotherapy	with family	5	5	5	5

Targeted therapy	with family	1	1	1	5
surgery	with family	1	1	1	5
Targeted therapy	with family	3	3	5	5
Targeted therapy	with family	1	1	1	3
surgery	with family	1	1	5	5
Chemotherapy	with family	5	5	5	5
Chemotherapy	with family	2	2	2	4
surgery	with family	1	1	1	5
Chemotherapy	with family	1	1	2	4
Targeted therapy	with family	1	1	1	5
surgery	with family	1	1	1	1
Chemotherapy	with family	5	1	1	5
Chemotherapy	with family	1	1	1	5
Chemotherapy	alone	1	1	1	3
surgery	with family	1	1	1	1
surgery	with family	2	3	2	5
Chemotherapy	with family	1	1	5	5

Q5	Q6	Q7	Q8	Q9	Q10	Q11
5	5	5	5	5	5	5
3	3	5	5	5	4	5
4	3	5	5	5	5	5
1	1	4	4	4	4	1
4	4	3	4	4	4	4
5	5	5	5	5	5	5
5	5	5	5	5	5	5
4	5	5	5	5	4	5
2	5	4	2	4	5	1
5	5	5	5	5	5	3
1	5	2	4	5	5	4
5	4	5	5	5	5	2
4	5	5	5	5	5	4
4	4	4	5	5	5	5
2	2	5	5	5	4	3
5	5	4	5	5	5	5
3	5	5	5	5	5	5
5	5	5	5	5	5	1
5	5	5	5	5	5	5
5	1	5	5	5	1	5
2	4	2	2	4	2	4
1	1	5	5	5	5	1
1	4	4	4	4	4	1
2	4	3	3	4	3	3
4	4	4	4	5	5	5
1	5	5	5	5	5	5
3	3	4	4	3	4	3
4	4	5	5	5	5	5
3	4	1	3	2	5	2
5	5	5	5	5	5	5
1	1	1	1	1	5	1
4	4	5	5	5	5	5
1	1	5	5	5	5	5
5	5	1	1	5	5	5
1	1	5	5	5	5	5
1	1	5	5	5	5	5
1	1	2	5	5	4	4
4	4	5	5	5	5	5
5	5	4	5	5	5	5
4	4	5	5	4	4	5
4	4	5	5	5	5	5
3	3	5	5	5	5	5
5	5	5	1	5	4	5
5	5	1	5	5	5	5
1	1	1	5	5	5	1
4	4	5	5	5	5	5
5	5	5	5	5	5	5
4	4	5	5	5	5	5
1	1	4	5	5	5	5
5	5	5	5	5	5	5
5	5	5	5	5	5	5
5	5	5	5	5	5	5

5	5	5	5	5	5	5
1	1	5	5	5	5	5
5	5	5	5	5	5	5
5	5	2	4	5	5	1
1	1	5	5	5	5	5
5	5	5	5	5	5	5
4	4	5	5	5	4	4
1	1	5	5	5	5	4
4	4	4	4	4	4	4
1	1	5	5	5	5	5
1	1	5	5	5	5	5
5	5	5	5	5	5	5
1	1	5	5	5	5	5
1	3	1	1	3	3	4
1	2	1	5	3	4	2
2	5	5	5	5	5	4
1	1	1	5	5	5	1

Q12	Q13	Q14	Q15	Q16	Q17	Q18
5	5	5	5	5	5	5
4	4	5	5	5	5	2
4	5	5	5	5	5	5
1	1	3	3	5	5	5
2	2	4	4	4	4	4
5	5	5	5	5	5	5
5	5	5	5	5	5	5
4	4	4	4	5	4	5
2	4	2	2	1	2	2
4	4	4	4	4	4	5
1	1	4	1	4	1	4
5	5	5	5	4	5	5
4	4	3	5	5	5	3
4	3	4	4	4	5	5
4	5	5	5	5	5	4
5	5	5	5	5	5	5
5	5	5	5	5	5	5
5	5	5	5	5	5	5
5	5	5	5	5	5	5
1	1	1	1	5	5	1
2	2	4	2	4	4	4
1	1	5	1	5	5	5
1	1	1	1	4	1	1
3	3	3	3	4	4	3
1	1	1	1	1	1	1
1	1	1	1	1	1	1
2	3	3	3	4	3	2
5	5	5	5	5	5	5
1	1	1	2	1	1	1
5	5	5	5	5	5	4
5	5	5	5	5	5	1
5	5	5	5	5	5	5
2	2	2	2	2	2	2
5	5	5	5	5	5	5
1	1	1	1	1	1	1
4	2	2	2	2	2	2
5	5	5	5	5	5	5
5	5	5	5	3	1	1
4	4	4	5	5	5	4
4	4	5	4	5	5	4
5	3	3	3	3	3	3
4	4	4	4	4	4	4
5	5	5	5	5	5	4
5	5	5	5	5	5	5
1	1	5	5	1	5	1
4	4	3	5	5	5	5
5	5	5	5	5	5	5
5	5	5	5	5	5	5
1	1	1	1	1	1	1
4	4	4	5	4	3	3
4	4	4	5	5	3	4
5	5	5	5	5	5	5

1	1	1	4	4	3	3
4	4	4	2	2	2	2
4	4	4	5	4	4	4
1	5	5	5	5	5	5
5	5	4	4	4	3	3
5	5	5	5	5	5	5
5	5	4	5	5	5	5
5	5	5	5	5	3	3
5	4	5	5	5	5	4
4	4	4	5	5	4	5
1	1	1	1	5	5	1
1	1	1	1	1	5	1
5	5	5	5	5	5	5
2	5	5	5	5	5	5
5	5	5	5	5	5	1
5	5	5	5	5	5	5
1	1	5	5	1	5	1

Q19	Q20	Q21	Q22	Q23	Q24	Q25
5	5	5	5	5	5	5
5	5	5	5	5	5	5
5	5	5	5	5	5	5
5	5	5	5	5	5	5
4	4	4	4	5	4	4
5	5	5	5	5	1	1
5	5	5	5	5	5	5
5	5	5	5	5	4	5
2	2	4	5	2	5	5
5	5	5	5	4	5	5
4	5	4	5	5	5	5
5	4	5	5	4	2	5
3	5	5	4	5	5	5
5	4	4	4	4	4	4
5	5	1	4	5	5	4
5	5	5	5	5	5	5
5	5	5	5	5	5	5
5	5	5	5	5	5	5
5	5	5	5	5	4	4
1	5	1	1	1	1	1
4	4	4	4	4	4	5
5	5	5	5	1	5	1
1	3	1	4	1	4	1
1	4	4	4	3	3	4
1	1	1	1	1	1	3
1	1	5	5	1	5	1
3	3	3	3	2	2	3
5	5	5	5	5	5	5
1	1	1	4	1	1	1
5	5	4	3	5	5	5
1	1	1	5	1	5	1
5	5	4	4	5	5	5
2	2	2	2	5	5	5
5	5	5	5	5	5	5
1	1	1	5	4	3	5
2	2	2	2	4	4	4
5	5	5	5	5	5	5
1	1	1	1	5	5	5
5	5	5	5	5	2	5
5	5	5	5	5	5	5
3	3	3	3	5	3	5
4	3	3	3	3	5	3
4	4	4	4	5	5	5
5	5	1	5	5	5	1
1	5	5	5	1	1	5
5	5	5	5	5	5	5
5	5	5	5	5	5	5
5	5	5	5	5	4	5
1	1	1	1	1	5	5
3	3	3	3	3	5	5
4	4	4	4	5	5	4
5	5	1	5	5	5	1

3	3	3	4	5	5	5
2	2	2	2	5	5	5
4	4	4	5	5	5	4
1	5	1	5	5	5	1
3	3	5	4	5	5	4
5	5	5	5	5	5	5
5	5	4	4	4	4	4
4	5	4	3	5	5	5
5	5	5	4	4	4	4
4	4	4	4	5	5	3
1	5	5	5	5	5	5
1	1	1	1	1	1	1
5	5	5	5	5	3	5
5	5	5	5	5	5	1
5	5	5	5	3	5	5
5	5	4	4	4	5	3
1	5	5	5	1	1	5

Q26	Q27	Q28	Q29	Q30	Q31	Q32
5	5	5	5	5	5	5
5	5	5	5	5	5	5
5	5	5	5	5	5	5
5	5	4	3	3	4	5
4	4	4	4	4	1	4
5	5	5	5	5	1	5
5	5	5	5	5	1	5
4	4	5	4	4	4	5
2	5	5	4	4	4	4
5	5	5	5	5	5	5
5	4	4	5	1	4	4
5	5	4	1	1	1	5
5	5	5	5	3	5	5
4	4	4	4	4	4	5
5	5	4	5	4	5	3
5	5	5	5	5	5	5
5	5	5	5	5	5	5
5	4	5	5	5	5	5
4	4	5	5	5	4	4
1	5	5	5	5	5	1
5	4	4	4	4	4	4
5	5	5	1	1	5	5
1	1	1	1	1	1	1
4	4	4	4	3	3	3
1	1	5	5	5	5	5
1	1	1	1	1	1	1
2	3	3	3	3	3	3
5	5	5	5	5	4	5
2	1	3	3	3	3	1
5	5	5	3	3	3	5
5	5	5	5	5	1	5
5	5	5	5	5	3	5
5	1	1	1	1	3	5
5	3	3	3	3	3	5
5	1	4	5	5	5	5
3	2	2	5	5	5	5
4	4	5	5	5	5	5
5	1	5	1	1	1	5
5	5	5	5	4	4	5
5	4	4	3	3	3	5
3	2	5	3	3	3	5
3	3	5	5	5	5	5
5	4	5	5	5	5	5
5	5	5	5	5	5	5
1	5	1	1	1	1	1
5	4	5	5	5	5	5
5	5	5	1	1	1	5
4	5	5	5	5	5	5
5	1	5	5	5	5	5
5	5	3	3	3	3	5
4	2	2	5	5	5	5
5	5	5	5	5	5	5

5	1	1	1	1	1	5
1	1	5	5	5	5	5
4	3	3	3	3	3	5
5	5	5	5	5	5	5
3	3	5	5	5	5	5
5	5	5	5	5	5	5
5	5	4	4	4	4	4
5	1	5	5	5	5	5
4	5	5	4	1	1	5
4	1	5	1	1	1	5
5	5	5	5	5	5	5
1	1	1	1	1	1	1
5	5	5	5	5	5	5
5	5	5	1	1	1	5
5	5	5	1	1	5	5
3	4	4	5	5	4	5
1	5	1	1	1	1	1

Q33	Q34	Q35	Q36	Q37	Q38	Q39
5	5	5	5	5	5	5
4	4	5	4	4	3	3
5	5	5	5	5	5	5
5	5	5	5	5	5	4
4	4	4	4	2	4	3
5	5	5	1	1	1	1
5	5	5	5	5	5	5
4	4	5	4	1	4	4
2	4	4	2	1	4	1
5	5	5	5	4	5	5
5	5	1	1	1	1	4
5	4	5	5	4	5	5
5	5	4	4	5	5	5
5	5	5	4	4	4	4
5	5	4	4	4	5	4
5	5	5	5	5	5	5
5	5	5	5	5	5	5
5	5	3	3	5	4	3
5	5	3	4	3	3	4
1	1	3	1	1	1	1
5	5	1	2	4	4	4
5	5	1	1	1	1	1
1	1	1	1	1	1	1
4	3	3	3	1	3	4
5	5	4	1	1	5	1
1	1	1	1	1	1	1
3	3	4	3	3	2	2
5	5	5	4	1	4	4
1	5	1	1	1	1	1
5	4	5	3	3	3	3
5	5	1	1	1	1	1
5	5	5	4	3	3	3
4	5	4	4	3	4	3
5	5	5	5	3	4	4
4	4	5	3	1	4	1
3	3	4	3	3	4	2
5	5	4	3	2	5	1
5	5	5	4	1	5	5
3	3	5	3	3	4	3
5	4	5	4	3	4	3
5	5	5	5	1	4	4
5	4	5	3	3	3	3
5	5	5	1	1	1	1
5	5	5	5	5	5	5
5	1	1	1	1	1	1
5	5	5	5	5	5	4
5	5	5	5	5	5	3
5	4	4	4	2	4	2
5	5	5	5	1	5	5
5	5	5	5	2	5	4
5	5	5	4	2	5	2
5	5	1	1	5	5	5

5	5	5	5	3	5	5
5	5	2	2	2	5	3
5	4	5	5	2	5	5
5	4	1	2	2	5	1
5	4	3	3	2	4	2
5	5	5	5	5	5	5
4	4	4	4	2	4	4
5	5	5	5	1	5	5
1	1	3	3	3	3	1
5	5	5	1	1	1	5
5	5	5	5	5	5	5
1	1	1	1	1	1	1
5	5	5	5	1	5	1
5	5	5	5	5	5	5
5	5	1	3	5	5	1
4	4	3	5	2	5	1
5	1	1	1	1	1	1

Q40	Q41	Q42	Q43	Q44	Q45	Q46
5	5	5	5	5	5	5
3	4	3	3	3	5	5
5	5	5	5	5	5	5
4	3	3	3	3	4	3
3	4	4	4	4	4	4
1	5	5	5	5	5	5
1	5	5	5	5	4	5
4	5	4	4	4	4	1
2	1	2	1	5	5	5
5	5	5	5	5	5	5
1	4	1	1	5	5	5
2	4	4	3	5	5	4
4	3	5	5	5	5	5
4	4	4	4	4	3	4
3	3	3	2	5	5	5
5	3	5	5	2	5	5
5	5	5	5	5	5	5
3	2	3	3	3	4	4
2	4	1	3	3	4	4
1	1	1	1	1	5	1
3	4	4	2	4	2	4
1	1	1	1	1	5	5
1	1	1	1	1	4	1
3	3	3	3	3	5	4
1	1	2	2	2	4	4
1	1	1	1	1	1	1
3	3	3	3	2	4	3
3	3	2	2	5	5	5
1	1	1	1	3	5	5
3	3	3	3	4	5	5
1	1	1	1	5	5	5
3	3	3	3	3	5	5
3	3	3	3	3	3	3
1	1	1	1	4	4	3
1	1	1	5	5	5	1
2	2	2	2	3	5	5
1	1	1	5	4	5	5
5	5	3	5	5	5	5
3	3	3	3	4	3	3
2	2	2	2	3	4	3
4	4	3	4	4	5	5
2	2	2	3	4	5	5
1	1	1	1	5	5	5
5	5	5	5	5	5	5
1	1	1	1	1	1	5
4	5	4	3	3	5	5
3	2	2	4	5	5	5
2	2	2	2	3	5	5
1	1	1	5	5	5	5
3	4	3	5	5	4	4
2	2	2	4	4	5	5
5	1	5	5	5	5	1

2	2	2	2	5	3	3
2	3	1	3	5	5	4
2	2	2	5	5	5	5
3	1	1	1	3	5	5
2	2	2	1	3	5	3
5	5	5	5	5	5	5
4	3	4	4	2	4	4
1	4	3	3	5	5	5
1	1	1	1	3	3	3
1	4	1	1	5	5	5
5	5	5	5	5	5	5
1	1	1	1	1	1	1
1	3	1	5	5	5	5
5	5	5	1	5	1	5
1	3	3	1	5	5	5
1	1	1	1	3	5	5
1	1	1	1	1	1	5

PQ1	PQ2	PQ3	PQ4	PQ5	PQ6	PQ7
3	3	3	3	3	3	5
3	3	4	5	3	3	5
1	1	1	5	1	1	5
2	2	2	2	1	1	4
3	3	4	3	2	3	5
5	5	5	5	1	5	5
1	1	1	1	1	1	5
1	1	2	3	2	2	4
1	1	1	4	1	3	5
1	1	1	1	1	1	1
1	1	1	1	2	4	2
5	5	1	1	1	1	5
1	1	1	5	1	5	5
1	1	1	1	1	1	5
4	1	1	1	1	1	4
2	1	1	1	1	1	4
1	1	1	1	1	1	4
3	2	2	4	2	2	4
2	2	2	4	2	2	5
1	1	1	1	1	1	4
1	1	1	1	1	1	5
1	1	1	3	1	1	5
1	1	1	1	1	1	1
3	3	3	4	3	3	3
5	1	5	5	5	5	5
1	1	1	1	1	1	1
2	2	2	3	3	3	5
2	2	2	5	2	2	5
1	1	1	2	1	1	2
#NULL!	#NULL!	#NULL!	#NULL!	#NULL!	#NULL!	#NULL!
1	1	1	5	2	2	2
1	1	4	5	3	3	5
1	1	1	1	1	1	3
1	1	1	1	1	1	4
1	1	1	5	1	1	1
1	1	1	1	1	1	3
1	1	1	5	1	1	1
1	1	1	5	1	1	5
1	1	1	4	1	1	5
4	4	1	4	1	1	4
1	1	1	1	1	1	3
1	1	1	3	1	1	1
1	1	1	5	1	1	5
1	1	1	1	1	1	5
2	2	2	4	3	3	4
1	1	1	1	3	3	4
1	1	1	1	1	1	4
1	1	1	1	1	1	1
1	1	1	5	1	1	5
1	1	1	1	5	5	5
1	1	1	5	1	1	5
1	1	1	1	1	1	3

1	1	4	5	1	1	4
1	1	1	5	1	1	5
1	1	1	5	1	1	1
1	1	5	5	5	5	5
1	1	1	1	2	2	4
1	1	1	1	1	1	1
#NULL!	#NULL!	#NULL!	#NULL!	#NULL!	#NULL!	#NULL!
1	1	1	5	1	1	4
1	1	1	3	1	1	5
1	1	1	1	1	1	4
1	1	1	5	1	1	5
1	1	1	1	1	1	5
1	1	1	1	1	3	1
5	1	5	1	1	1	5
5	1	5	5	1	1	5
2	4	3	5	5	5	4
2	2	2	4	3	3	4

PQ8	PQ9	PQ10	PQ11	PQ12	PQ13	PQ14
5	5	4	2	1	1	1
5	5	5	5	2	2	4
5	5	5	5	5	5	5
4	4	4	4	1	1	1
4	4	5	4	3	4	5
5	5	5	5	1	1	5
5	5	5	1	4	4	4
4	4	4	1	1	1	1
2	5	4	1	1	1	1
1	1	1	1	1	1	1
5	4	2	2	1	1	1
5	3	2	1	1	5	5
5	5	5	5	1	3	5
1	1	1	1	1	1	1
4	4	4	4	1	1	4
4	4	5	1	1	1	1
4	4	4	1	1	1	1
5	3	5	2	2	2	2
5	5	4	1	1	1	2
3	5	3	1	1	1	1
5	5	1	5	1	1	1
4	4	5	1	1	1	1
1	1	1	1	1	1	1
3	3	3	3	3	3	3
5	5	5	1	1	1	1
4	1	4	1	1	1	1
5	5	3	3	3	3	3
5	5	2	2	2	2	2
2	1	3	3	1	1	1
#NULL!	#NULL!	#NULL!	#NULL!	#NULL!	#NULL!	#NULL!
2	2	5	1	5	5	5
5	3	5	1	1	1	1
4	3	3	1	1	1	1
4	1	1	1	1	1	1
5	1	5	1	1	1	1
4	3	4	1	1	1	1
5	1	5	1	1	1	5
5	5	5	1	1	1	1
5	1	5	1	1	1	1
5	4	4	1	1	1	4
5	1	5	1	1	1	1
4	4	5	1	1	1	1
5	5	5	5	1	1	1
5	5	5	1	1	1	1
4	4	4	1	1	1	5
4	4	4	4	1	1	1
5	1	1	1	1	1	1
5	1	1	1	1	1	5
5	1	1	1	1	1	1
1	1	1	1	5	5	5
4	1	1	1	1	1	1
3	1	5	1	1	1	1

5	5	5	1	1	1	1
5	3	3	1	1	1	1
5	1	1	1	1	1	1
5	5	5	1	1	1	5
5	1	5	1	1	1	1
5	1	5	1	1	1	1
#NULL!	#NULL!	#NULL!	#NULL!	#NULL!	#NULL!	#NULL!
5	4	5	1	1	1	1
5	4	4	1	1	1	5
5	1	1	1	1	1	1
1	1	1	1	1	1	5
5	5	5	1	1	1	5
1	1	1	1	1	1	5
1	5	1	1	1	1	1
5	5	5	1	1	1	1
5	1	1	1	1	1	5
4	4	4	1	1	1	5

PQ15	PQ16	PQ17	PQ18	PQ19	PQ20	PQ21	
1	5	5	2	5	4	2	
3	3	3	3	3	3	5	
5	5	5	3	5	1	5	
1	2	4	3	2	2	2	
4	3	4	4	4	4	4	
5	5	5	5	5	5	1	
4	4	3	4	3	4	3	
1	1	1	1	1	1	1	
2	2	2	2	2	2	2	
1	1	3	3	3	3	3	
1	1	4	2	2	2	1	
2	5	5	2	2	2	2	
1	5	5	1	5	5	5	
1	1	1	1	1	1	1	
1	4	1	1	1	4	4	
1	1	1	1	1	1	1	
1	1	4	1	1	1	1	
2	2	2	5	5	5	2	
2	2	5	3	3	3	2	
1	1	1	1	1	1	1	
1	1	1	1	1	1	1	
1	1	2	2	2	2	2	
1	1	1	1	1	1	1	
3	3	3	3	3	3	3	
1	1	1	1	1	1	1	
1	1	1	1	1	1	1	
3	3	3	3	3	3	3	
2	2	2	2	2	2	2	
1	1	2	1	1	1	1	
#NULL!	#NULL!	#NULL!	#NULL!	#NULL!	#NULL!	#NULL!	
5	4	4	3	1	1	1	
1	1	1	1	1	1	1	
1	1	1	1	1	1	1	
1	1	1	1	1	1	1	
1	1	1	1	1	1	1	
1	1	1	1	1	1	1	
1	1	1	1	1	1	1	
5	4	1	1	1	1	1	
1	1	1	1	1	1	1	
1	1	5	1	1	1	1	
1	1	1	1	1	1	1	
1	1	1	1	1	1	1	
1	1	1	1	1	1	1	
1	1	4	1	3	4	1	
1	1	4	1	1	1	1	
1	4	5	1	1	1	1	
1	1	1	1	1	1	1	
1	1	1	1	1	1	1	
1	1	1	1	1	1	1	
5	5	5	5	5	5	5	
1	1	1	1	1	1	1	
1	1	1	1	1	1	1	

1	1	1	1	1	1	1
1	1	1	1	1	4	1
1	1	1	1	1	1	1
5	5	5	5	5	5	1
1	1	1	1	1	1	1
1	1	1	1	1	1	1
#NULL!	#NULL!	#NULL!	#NULL!	#NULL!	#NULL!	#NULL!
1	1	1	1	1	1	1
1	1	1	1	1	1	1
1	1	1	1	1	1	1
1	1	1	1	1	1	1
1	1	1	1	3	1	1
1	1	1	1	1	1	1
1	1	1	1	1	1	1
5	5	1	5	5	5	5
1	1	1	1	1	1	1
1	1	4	1	1	1	1

PQ22	PQ23	PQ24	PQ25	PQ26	PQ27	PQ28
5	5	5	4	4	3	3
3	3	5	3	3	3	3
5	1	1	5	1	5	5
2	2	2	2	2	2	2
5	5	5	4	5	4	5
5	5	5	5	5	1	1
4	3	1	1	1	1	1
1	1	4	2	2	1	1
2	2	1	2	2	1	1
3	3	3	3	3	3	3
1	1	5	1	1	1	1
3	2	1	1	1	5	4
5	5	1	5	5	1	1
1	1	1	1	1	1	1
4	4	4	1	4	1	1
1	1	1	1	1	1	1
4	1	1	1	1	1	1
5	2	1	2	2	2	2
5	2	5	3	3	3	3
1	1	5	1	1	1	1
1	1	1	1	1	1	1
2	2	5	4	2	2	2
1	1	1	1	1	1	1
3	3	3	3	3	3	3
1	1	1	1	1	1	5
1	1	1	4	1	1	1
3	3	1	4	1	1	1
2	2	5	3	3	2	2
1	2	3	2	1	1	2
#NULL!	#NULL!	#NULL!	#NULL!	#NULL!	#NULL!	#NULL!
1	1	1	1	1	5	5
1	1	5	1	1	1	1
1	1	1	1	1	1	1
1	1	1	1	1	1	1
1	1	1	1	1	1	1
1	1	1	1	1	1	1
1	1	5	1	1	1	5
1	1	1	1	1	1	1
1	1	1	1	1	1	5
1	1	1	1	1	1	1
1	1	1	5	1	1	5
1	1	1	1	1	1	1
1	5	1	1	1	1	5
1	1	5	4	4	1	1
1	1	1	1	1	1	1
1	1	1	1	1	1	1
1	1	1	1	1	1	1
1	1	5	1	1	1	1
1	1	1	1	1	1	1
5	5	1	4	5	5	5
1	1	1	2	1	1	4
1	1	1	1	1	5	1

1	1	1	5	1	1	1
1	1	1	1	1	1	1
1	1	1	1	1	1	1
5	1	1	1	1	1	1
1	1	5	1	1	1	2
1	1	5	1	1	1	1
#NULL!	#NULL!	#NULL!	#NULL!	#NULL!	#NULL!	#NULL!
1	1	5	5	1	1	5
5	1	1	1	1	1	1
1	1	1	5	1	1	1
1	1	5	1	1	1	1
1	1	1	1	1	1	4
1	1	5	4	1	1	1
1	1	1	5	1	1	1
5	1	5	1	1	1	5
1	1	5	3	1	4	4
1	1	1	1	1	1	1

PQ29	PQ30	PQ31	PQ32	PQ33	PQ34	PQ35
3	1	1	5	5	5	2
3	3	3	4	4	4	4
5	5	5	5	5	5	5
2	2	2	2	2	2	2
4	4	4	3	3	3	4
1	1	1	1	1	5	1
1	1	1	5	1	1	1
1	1	1	1	2	2	2
1	1	1	2	2	2	2
3	3	3	3	3	3	3
1	1	1	1	1	1	1
4	2	2	5	5	5	1
1	1	5	5	5	5	5
1	1	1	4	4	4	1
1	4	4	4	4	4	5
1	1	1	1	1	1	1
1	1	1	1	1	1	1
2	2	2	3	3	3	2
3	3	3	3	5	5	1
1	1	1	1	1	1	1
1	1	1	4	1	1	1
2	2	2	4	4	2	1
1	1	1	1	1	1	1
3	3	3	3	3	3	4
1	1	1	5	1	5	1
1	1	1	1	1	1	1
1	1	1	3	4	4	2
2	2	2	2	4	4	2
1	1	2	1	1	1	2
#NULL!	#NULL!	#NULL!	#NULL!	#NULL!	#NULL!	#NULL!
4	1	1	5	4	3	2
1	1	1	1	4	4	1
1	1	1	5	1	1	1
1	1	1	4	2	2	1
1	1	1	1	1	1	1
1	1	1	4	3	3	2
1	1	1	1	1	5	1
1	1	1	5	1	1	5
1	1	1	1	5	1	1
1	1	1	1	1	1	1
1	1	1	1	1	1	1
1	1	5	5	1	1	1
1	1	5	1	1	1	1
1	1	1	4	4	4	4
1	1	1	4	4	3	1
1	1	1	5	5	5	1
1	1	1	5	1	4	1
1	1	1	5	1	1	1
1	1	1	5	1	1	1
5	3	3	5	5	5	1
1	1	1	1	1	1	1
1	1	1	5	1	1	1

1	1	1	5	1	5	1
1	1	1	4	1	1	1
1	1	1	1	1	1	1
1	1	1	5	5	5	1
1	1	1	3	1	1	1
1	1	1	1	1	1	1
#NULL!	#NULL!	#NULL!	#NULL!	#NULL!	#NULL!	#NULL!
1	1	1	5	5	5	1
1	1	1	4	1	1	1
1	1	1	1	1	5	1
1	1	1	1	5	1	1
1	1	1	5	5	1	1
1	1	1	4	1	1	1
1	1	1	5	5	5	4
1	1	1	5	5	5	1
1	1	1	4	4	4	1
1	1	1	4	4	3	1

PQ36	PQ37	PQ38	PQ39	PQ40	PQ41	PQ42
2	2	2	2	2	2	2
3	3	3	3	3	3	3
5	3	3	1	1	2	2
2	2	2	2	2	2	2
3	3	2	2	2	2	2
1	5	5	5	1	1	1
1	1	1	1	1	1	1
2	2	2	2	2	2	2
2	1	2	2	2	1	1
3	3	3	3	3	3	3
1	1	1	1	1	1	1
2	5	1	1	5	1	4
5	5	5	5	5	1	3
1	1	1	1	1	1	1
5	1	5	5	5	3	5
1	1	1	1	1	1	1
1	1	1	1	1	1	1
2	3	4	2	2	2	2
3	5	3	3	3	5	3
1	1	1	1	1	1	1
1	5	5	5	1	1	1
1	1	1	1	1	1	1
1	1	1	1	1	1	1
3	3	3	3	3	3	3
1	1	1	1	1	1	1
1	1	1	1	1	1	1
2	4	2	2	2	3	2
2	3	2	2	2	2	2
1	2	1	1	2	1	1
#NULL!	#NULL!	#NULL!	#NULL!	#NULL!	#NULL!	#NULL!
1	1	1	1	1	1	1
1	1	1	1	1	1	1
1	4	1	1	1	1	1
1	1	5	1	1	1	1
1	1	1	1	1	1	1
1	1	1	1	1	1	1
1	1	1	1	1	1	1
1	5	1	1	1	1	1
1	1	1	1	1	1	1
1	1	1	1	1	1	1
1	1	1	1	1	1	1
1	1	1	1	1	1	1
1	1	1	1	1	1	1
3	3	3	3	3	3	3
1	4	1	1	1	1	1
1	3	1	1	1	1	1
1	3	4	1	1	1	1
1	1	1	1	1	1	1
1	1	1	4	1	1	1
5	4	4	5	1	1	1
1	1	1	1	1	1	1
1	1	1	1	1	1	1

1	1	1	1	1	1	1
1	1	1	1	1	1	1
1	3	1	1	1	1	1
1	5	1	5	5	5	1
1	1	1	1	1	1	1
1	1	1	1	1	1	1
#NULL!	#NULL!	#NULL!	#NULL!	#NULL!	#NULL!	#NULL!
1	1	4	1	1	1	1
1	1	1	1	1	1	1
1	1	1	1	1	1	1
1	1	1	1	1	1	1
1	4	1	1	1	1	1
1	1	1	1	1	1	1
4	5	1	5	1	1	1
1	5	1	1	1	1	1
1	3	3	1	1	1	1
1	4	1	1	1	1	1

PQ43	PQ44	PQ45	PQ46	examination_Time1	disease_Time1
2	2	2	2	5.00	5.00
3	3	4	4	3.17	4.80
2	5	1	5	3.33	5.00
2	2	2	2	3.00	3.40
2	3	3	3	2.83	3.80
1	1	5	1	3.83	5.00
1	1	1	1	5.00	5.00
2	2	4	2	3.33	4.80
1	1	2	2	2.83	3.20
3	3	3	3	5.00	4.60
1	3	3	1	3.67	4.00
1	1	1	1	3.50	4.40
1	5	5	1	4.17	4.80
1	1	1	1	2.00	4.80
5	5	5	5	3.17	4.40
1	1	4	1	4.00	4.80
1	1	1	1	3.33	5.00
2	4	2	2	4.67	4.20
3	1	1	1	5.00	5.00
1	1	4	1	1.67	4.20
1	1	1	1	3.17	2.80
1	1	5	1	1.67	4.20
1	1	1	1	1.50	3.40
3	3	4	4	3.00	3.20
1	1	5	1	3.00	4.60
1	1	4	1	1.67	5.00
2	2	4	4	3.00	3.60
2	3	4	2	3.17	5.00
2	1	1	2	2.17	2.60
#NULL!	#NULL!	#NULL!	#NULL!	5.00	5.00
1	4	3	1	1.00	1.80
1	1	4	1	3.33	5.00
1	1	1	1	2.33	5.00
1	1	1	1	3.00	3.40
1	1	1	1	1.67	5.00
1	3	3	1	3.17	5.00
5	4	5	1	2.17	4.00
1	1	5	1	3.67	5.00
1	1	1	1	4.00	4.80
1	1	1	1	3.83	4.60
1	1	5	1	4.00	5.00
1	1	1	1	3.50	5.00
1	1	5	1	2.83	4.00
4	4	4	3	3.50	4.20
1	1	1	1	2.33	3.40
1	2	5	1	3.50	5.00
1	4	3	1	2.33	5.00
1	1	5	1	4.67	5.00
1	5	5	1	1.00	4.80
1	1	1	5	3.33	5.00
1	3	4	1	2.33	5.00
1	3	3	1	5.00	5.00

1	1	5	1	3.00	5.00
1	5	1	1	1.67	5.00
1	1	1	1	4.33	5.00
5	1	5	5	2.67	3.40
1	4	1	1	2.33	5.00
1	1	5	5	5.00	5.00
#NULL!	#NULL!	#NULL!	#NULL!	3.00	4.60
1	4	4	1	1.67	4.80
1	1	1	1	2.67	4.00
1	3	4	1	1.67	5.00
1	1	5	1	1.00	5.00
1	5	5	1	3.67	5.00
1	1	5	1	1.67	5.00
1	5	5	1	1.67	2.40
1	5	5	1	1.17	3.00
1	1	1	1	3.17	4.80
1	1	1	1	2.33	3.40

treatment_Time1	physical_Time1	psychosocial_Time1	examination_Time2	disease_Time2
5.00	5.00	5.00	3.00	4.20
4.67	4.86	3.77	3.50	5.00
4.93	5.00	5.00	1.67	5.00
4.20	4.14	4.00	1.67	4.00
3.80	3.57	3.69	3.00	4.40
4.47	4.43	3.46	4.33	5.00
5.00	4.43	4.62	1.00	4.20
4.53	4.29	3.69	1.83	3.40
2.80	4.00	2.85	1.83	3.40
4.53	5.00	4.92	1.00	1.00
3.60	3.86	2.69	1.67	3.00
4.60	3.14	4.23	2.33	3.20
4.40	4.71	4.62	2.33	5.00
4.13	4.29	4.08	1.00	1.80
4.47	4.43	4.00	1.50	4.00
5.00	5.00	4.62	1.17	3.60
5.00	5.00	5.00	1.00	3.40
5.00	4.86	3.46	2.50	3.80
4.80	4.57	3.31	2.33	4.00
1.80	3.86	1.46	1.00	3.20
3.73	4.14	3.31	1.00	4.20
3.67	3.86	1.92	1.33	3.80
1.73	1.00	1.23	1.00	1.00
3.33	3.57	3.15	3.17	3.00
1.13	4.43	2.54	4.33	4.20
1.80	1.00	1.00	1.00	2.20
2.73	3.00	2.92	2.50	4.20
5.00	4.86	3.69	2.50	3.80
1.33	2.14	2.08	1.17	2.20
4.73	4.14	3.62	#NULL!	#NULL!
3.40	4.43	2.23	2.00	2.40
4.87	4.71	3.69	2.83	3.80
2.80	2.29	3.38	1.00	2.80
5.00	3.57	3.15	1.00	2.20
2.13	4.14	2.85	1.67	2.60
2.60	3.86	3.08	1.00	3.00
4.93	4.86	3.23	1.67	2.60
3.27	2.71	4.46	1.67	4.20
4.53	4.43	3.31	1.50	3.40
4.73	3.86	3.15	2.50	3.60
3.40	3.71	4.08	1.00	3.00
3.67	4.71	3.38	1.33	3.00
4.67	4.86	2.54	1.67	5.00
4.47	5.00	5.00	1.00	4.20
2.87	2.14	1.31	2.67	3.40
4.73	4.86	4.46	1.67	4.00
5.00	3.29	4.15	1.00	2.40
4.87	5.00	3.15	1.00	1.80
1.80	4.43	3.77	1.67	2.60
3.80	3.86	4.15	2.33	1.80
4.20	4.14	3.62	1.67	2.40
4.47	5.00	3.77	1.00	2.60

3.33	2.14	3.62	2.17	4.00
2.93	4.43	3.23	1.67	3.40
4.27	3.57	4.00	1.67	1.80
3.93	5.00	2.62	3.67	4.20
4.00	4.71	2.77	1.33	3.20
5.00	5.00	5.00	1.00	2.60
4.60	4.14	3.62	#NULL!	#NULL!
4.47	4.43	4.00	1.67	3.80
4.53	3.14	2.08	1.33	3.80
4.27	2.71	3.08	1.00	2.40
3.40	5.00	5.00	1.67	1.80
1.27	1.00	1.00	1.00	4.20
4.87	5.00	3.62	1.33	1.00
4.53	3.29	4.38	2.33	2.60
4.60	3.86	3.31	3.00	4.20
4.53	4.43	2.85	4.00	2.40
2.87	2.14	1.31	2.67	3.40

treatment_Time2	physical_Time2	phychosocial_Time2
3.33	3.00	2.23
3.20	3.29	3.31
3.80	5.00	3.08
1.93	2.00	2.00
4.20	3.86	2.62
4.20	1.00	2.54
3.13	1.57	1.00
1.33	1.14	2.15
1.73	1.29	1.62
2.33	3.00	3.00
1.67	1.00	1.31
2.60	3.86	2.23
3.80	2.71	3.92
1.00	1.86	1.23
2.60	2.71	4.46
1.00	1.00	1.23
1.40	1.00	1.00
2.73	2.29	2.46
2.80	3.29	2.85
1.27	1.00	1.23
1.00	1.43	1.92
2.00	2.57	1.38
1.00	1.00	1.00
3.00	3.00	3.23
1.00	2.14	1.62
1.20	1.00	1.23
2.80	1.71	2.69
2.33	2.29	2.46
1.33	1.29	1.38
#NULL!	#NULL!	#NULL!
2.60	3.57	1.62
1.27	1.43	1.46
1.00	1.57	1.23
1.00	1.57	1.38
1.00	1.00	1.00
1.00	1.71	1.54
1.53	1.57	2.15
1.47	1.57	1.92
1.00	2.14	1.00
1.47	1.00	1.00
1.27	1.57	1.31
1.00	2.14	1.00
1.27	2.14	1.31
2.20	1.86	3.38
1.47	1.86	1.38
1.47	2.14	1.85
1.00	1.57	2.00
1.53	1.57	1.31
1.00	1.57	1.85
4.67	4.43	2.69
1.07	1.43	1.38
1.00	2.14	1.31

1.27	1.57	1.62
1.20	1.43	1.31
1.00	1.00	1.15
3.13	2.14	3.46
1.27	1.43	1.23
1.27	1.00	1.62
#NULL!	#NULL!	#NULL!
1.53	2.71	2.00
1.53	1.43	1.00
1.27	1.00	1.69
1.53	1.57	1.31
1.40	2.57	1.85
1.73	1.43	1.31
1.27	2.14	3.00
3.13	2.71	2.23
1.67	2.71	1.54
1.47	1.86	1.38
